# Changes in self-harm and suicide in California from 2017–2021: a population-based study

**DOI:** 10.21203/rs.3.rs-2395128/v1

**Published:** 2023-01-09

**Authors:** Julia J. Lund, Elizabeth Tomsich, Julia P. Schleimer, Veronica A. Pear

**Affiliations:** University of California Davis School of Medicine; University of California Davis School of Medicine; University of California Davis School of Medicine; University of California Davis School of Medicine

**Keywords:** Self-harm, Suicide, Health disparities, COVID-19, Gun violence

## Abstract

**Background:**

Self-harm and suicide are major public health problems with immediate and long-term effects on individuals, families, and communities. In 2020 and 2021, stressors wrought by the COVID-19 pandemic, stay-at-home mandates, economic turmoil, social unrest, and growing inequality likely modified risk for self-harm. The coinciding surge in firearm purchasing may have increased risk for firearm suicide. In this study, we examined changes in counts and rates of fatal and nonfatal intentional self-harm in California across sociodemographic groups during the first two years of the COVID-19 pandemic relative to prior years.

**Methods:**

We used California-wide death data and University of California (UC)-wide hospital data to summarize fatal and nonfatal instances of intentional self-harm across race/ethnicity, age, education, gender, region, and method of harm. We compared case counts and rates in 2020 and 2021 with 2017–2019 averages.

**Results:**

Suicide decreased overall in 2020 (4123 deaths; 10.5 per 100,000) and 2021 (4104; 10.4 per 100,000), compared to pre-pandemic (4484; 11.4 per 100,000). The decrease in counts was driven largely by males, white, and middle-aged Californians. Conversely, Black Californians and young people (age 10–19) experienced increased burden and rates of suicide. Firearm suicide also decreased following the onset of the pandemic, but relatively less than overall suicide; as a result, the proportion of suicides that involved a firearm increased (from 36.1% pre-pandemic to 37.6% in 2020 and 38.1% in 2021). Females, people aged 20–29, and Black Californians had the largest increase in the likelihood of using a firearm in suicide following the onset of the pandemic. Counts and rates of nonfatal, intentional self-harm in UC hospitals increased in 2020 (2160; 30.7 per 100,000) and 2021 (2175; 30.9 per 100,000) compared to pre-pandemic (2083; 29.6 per 100,000), especially among young people (age 10–19), females, and Hispanic Californians.

**Conclusions:**

The COVID-19 pandemic and co-occurring stressors coincided with heterogeneous changes in risk of self-harm and suicide across the California population. Marginalized racial groups, females, and younger people experienced increased risk for self-harm, particularly involving a firearm. Public health intervention and policy action are necessary to prevent fatal and nonfatal self-harm injuries and reduce related inequities.

## Background

On March 19, 2020, California went under a stay-at-home order in response to the COVID-19 pandemic.^1^ Non-essential businesses, such as bars, fitness clubs, and some stores were ordered to close, and residents were asked to “shelter-in-place” at home. Many people lost employment or had to leave jobs to caretake for children or family members. A high percentage of adults had trouble paying bills or rent due to the pandemic,^2^ and most became more socially isolated.^3^ At the same time, hundreds of thousands experienced loss of a loved one to COVID-19.^4^

Compounded with the pandemic and its repercussions were a variety of other co-occurring stressors in 2020 and 2021, including the widely publicized murder of George Floyd, subsequent protests and incidents of police brutality, political turmoil surrounding the 2020 presential election, climate-change-fueled megafires, and collective grief and trauma resulting from these events.^5, 6^ Notably, not all groups were equally impacted. Marginalized communities and racialized groups bore the disproportionate burden of the social, health, and economic consequences of 2020 and 2021^7^ as a result of the ongoing legacy of structural racism in the United States (U.S.), which has concentrated disadvantages (including poverty, under-funded schools, unemployment, over-policing and police violence, mass incarceration, and limited access to affordable healthcare, housing, and green spaces) among Black, Indigenous, and Hispanic communities.^8^

Financial difficulties, unemployment, social isolation, and trauma have all been linked to suicide and related risk factors (e.g., suicidality, depression).^9, 10^ One analysis found that in the years following the economic downturn from 2007 to 2009, an estimated 4,750 more Americans died by suicide than projected.^11^ A study of quarantined people during the severe acute respiratory syndrome (SARS) outbreak in 2003 found that approximately one-third of individuals experienced symptoms of depression and posttraumatic stress disorder (PTSD), with higher rates of PTSD symptoms associated with longer durations of quarantine.^12^ Prior pandemics have also been linked to suicide: some research suggests that deaths by suicide increased overall during the 1918–1919 influenza pandemic in the U.S.^13^ and among older people aged 65 and above in Hong Kong during the SARS epidemic.^14^

Several studies have examined the link between the COVID-19 pandemic and suicide, but findings are mixed. One 2021 survey of adults in the U.S. found an association between COVID-19-related experiences (i.e., general distress, fear of infection, effects of social distancing policies) and increased suicidal ideation and nonfatal suicide attempts, with a substantial proportion of those reporting suicidal ideation explicitly attributing it to COVID-19.^15^ Risk of self-harm has also been associated with high perceived stress due to COVID-19.^16^ However, a 2021 systematic review of time series analyses in Brazil, China, Ecuador, Mexico, Peru, Russian Federation, and Sri Lanka found no change in intentional self-harm during COVID-19.^17^ In the U.S., two studies in California found no change in intentional drug-related overdoses^18^ or suicidal ingestions reported to the California Poison Control system^19^ following the pandemic. At the same time, there is evidence that—contrary to expectations—deaths by suicide decreased in Cook County, Illinois and in four Texas counties through July 31, 2020.^20^ Differences between studies may stem in part from the populations under study. Importantly, analyses of aggregate trends may mask substantial heterogeneity in population subgroups, especially for subpopulations who comprise a minority of the overall population.^21^

Beginning in 2020, firearm and ammunition purchasing in the U.S. far surpassed expected levels. Through July 2020, there were an estimated 4.3 million excess firearm purchases nationally^22^ and, in California, approximately 110,000 people acquired a firearm and 390,000 purchased ammunition in response to the pandemic.^23^ Nationally, new firearm purchasers in 2020 and 2021 were more likely to be female, Black, or Hispanic.^24^ Handgun acquisition has been associated with large increases in firearm suicide risk, with a hazard ratio of nearly 8 among men and over 35 among women.^25^ Despite this purchasing surge and the high lethality of firearm suicide attempts,^26, 27^ few studies have examined pandemic-era trends in firearm suicide specifically, and none have looked at California, the most populous and diverse state in the U.S. In addition, most prior studies have failed to capture nonfatal suicide attempts and thus a more complete picture of the public health burden of intentional self-harm.

The current study examines deaths by suicide, deaths by firearm suicide, and hospital visits for nonfatal self-harm from 2017 through 2021 in California. Our aim is to assess changes in suicide and self-harm during the first two years of the COVID-19 pandemic and determine whether changes varied by sociodemographic groups. We assess both counts and rates to evaluate different dimensions of the problem. Counts indicate population burden by identifying groups with the highest number of injuries or deaths from self-harm, while rates allow for between-group comparisons and reveal disparities in how different groups experience suicide and self-harm.

## Methods

### Data

We used publicly available data on suicide from the California Department of Public Health – Vital Records Data (Cal-ViDa) query tool. The data contain statewide counts of deaths that occurred in California from January 1, 2017 to December 31, 2021, including information on decedents’ race and/or ethnicity (non-Hispanic white; non-Hispanic Black; non-Hispanic Asian; non-Hispanic Native American or Alaskan Native [AI/AN]; non-Hispanic Native Hawaiian or Pacific Islander [NH/PI]; Hispanic; or Other, which includes multi-race, other, and unknown), education level (Bachelor’s degree and higher or less than a Bachelor’s degree), gender identity (male or female), age (10 to 19, 20 to 29, 30 to 44, 45 to 64, 65+), county of residence (grouped into California Census Regions, displayed in Supplemental Fig. 1), and cause of death, coded using the International Classification of Disease, 10th Revision (ICD-10).^28^ Deaths by suicide were defined using ICD-10 codes for intentional self-harm by discharge of firearms (X72-X74) and by other and unspecified means (*U03, X60-X71, X75-X84, Y87.0).

Deidentified data on nonfatal self-harm injuries were provided by University of California (UC) Davis Health Informatics. The data included counts of initial outpatient, inpatient, or office visits with a self-harm related ICD-10 code,^29^ excluding subsequent encounters or those for sequelae, from all five UC Health hospitals: Davis, Irvine, Los Angeles, San Diego, and San Francisco. The data contained race/ethnicity (non-Hispanic white, non-Hispanic Black, non-Hispanic Asian, or Hispanic), sex assigned at birth (male or female), and age (10 to 19, 20 to 39, 40 to 59, 60+) of all patients.

As in prior research, given the small number of cases coded as suicide among people under the age of 10,^30^ we restricted both of our samples to people aged 10 years and older. We used race and Hispanic ancestry/origin (race/ethnicity) as proxies for sociocultural differences which may modify risk for self-harm^31, 32^ and for the effects of interpersonal and structural racism, including redlining, residential and social segregation, punitive immigration policy, mass incarceration, and the concentration and transmission of intergenerational trauma,^8, 33–38^ which are risk factors for self-harm.

### Analysis

We described rates and counts of fatal and nonfatal self-harm in California across our study period, comparing differences before and during the pandemic by method of fatal self-harm (firearm vs other) and across sociodemographic groups and geographic areas. Data on the method used in nonfatal self-harm injuries were not available. To calculate rates, we used population size estimates from the publicly available American Community Survey; 2017–2020 5-year estimates were used for all rate denominators, and the public use microdata sample was used for analyses on the subset of 10–19-year-olds since the microdata provide population counts disaggregated by race/ethnicity and gender for this age group. For suicide deaths, we used the statewide population aged 10 and older. For nonfatal self-harm, we used the population aged 10 and older of the city in which each hospital was located.

We compared annual counts and rates in 2020 and 2021 (which we refer to as “following the onset of the pandemic”) to the average of the 2017, 2018, and 2019 annual counts and rates (which we refer to as “pre-pandemic”). To produce estimates for suppressed small numbers (Cal-ViDa data indicate “<10” for all cell sizes 1–9 and UC Health data indicate “<11” for all cell sizes 0–10), we used a single imputation technique developed for public health data which uses data in years prior to or after a missing cell to inform replacement, and mean imputation by year for the remaining missing values.^39^ Up to 35% of Cal-ViDa observations had missing data (primarily due to suppressed county-level suicide counts in small counties) and up to 19% of UC Health observations had missing data (primarily due to suppressed self-harm counts among people aged 60+). All statistical analyses were done using R version 4.1.2.^40^

### Data

We used publicly available data on suicide from the California Department of Public Health – Vital Records Data (Cal-ViDa) query tool. The data contain statewide counts of deaths that occurred in California from January 1, 2017 to December 31, 2021, including information on decedents’ race and/or ethnicity (non-Hispanic white; non-Hispanic Black; non-Hispanic Asian; non-Hispanic Native American or Alaskan Native [AI/AN]; non-Hispanic Native Hawaiian or Pacific Islander [NH/PI]; Hispanic; or Other, which includes multi-race, other, and unknown), education level (Bachelor’s degree and higher or less than a Bachelor’s degree), gender identity (male or female), age (10 to 19, 20 to 29, 30 to 44, 45 to 64, 65+), county of residence (grouped into California Census Regions, displayed in Supplemental Fig. 1), and cause of death, coded using the International Classification of Disease, 10th Revision (ICD-10).^28^ Deaths by suicide were defined using ICD-10 codes for intentional self-harm by discharge of firearms (X72-X74) and by other and unspecified means (*U03, X60-X71, X75-X84, Y87.0).

Deidentified data on nonfatal self-harm injuries were provided by University of California (UC) Davis Health Informatics. The data included counts of initial outpatient, inpatient, or office visits with a self-harm related ICD-10 code,^29^ excluding subsequent encounters or those for sequelae, from all five UC Health hospitals: Davis, Irvine, Los Angeles, San Diego, and San Francisco. The data contained race/ethnicity (non-Hispanic white, non-Hispanic Black, non-Hispanic Asian, or Hispanic), sex assigned at birth (male or female), and age (10 to 19, 20 to 39, 40 to 59, 60+) of all patients.

As in prior research, given the small number of cases coded as suicide among people under the age of 10,^30^ we restricted both of our samples to people aged 10 years and older. We used race and Hispanic ancestry/origin (race/ethnicity) as proxies for sociocultural differences which may modify risk for self-harm^31, 32^ and for the effects of interpersonal and structural racism, including redlining, residential and social segregation, punitive immigration policy, mass incarceration, and the concentration and transmission of intergenerational trauma,^8, 33–38^ which are risk factors for self-harm.

### Analysis

We described rates and counts of fatal and nonfatal self-harm in California across our study period, comparing differences before and during the pandemic by method of fatal self-harm (firearm vs other) and across sociodemographic groups and geographic areas. Data on the method used in nonfatal self-harm injuries were not available. To calculate rates, we used population size estimates from the publicly available American Community Survey; 2017–2020 5-year estimates were used for all rate denominators, and the public use microdata sample was used for analyses on the subset of 10–19-year-olds since the microdata provide population counts disaggregated by race/ethnicity and gender for this age group. For suicide deaths, we used the statewide population aged 10 and older. For nonfatal self-harm, we used the population aged 10 and older of the city in which each hospital was located.

We compared annual counts and rates in 2020 and 2021 (which we refer to as “following the onset of the pandemic”) to the average of the 2017, 2018, and 2019 annual counts and rates (which we refer to as “pre-pandemic”). To produce estimates for suppressed small numbers (Cal-ViDa data indicate “<10” for all cell sizes 1–9 and UC Health data indicate “<11” for all cell sizes 0–10), we used a single imputation technique developed for public health data which uses data in years prior to or after a missing cell to inform replacement, and mean imputation by year for the remaining missing values.^39^ Up to 35% of Cal-ViDa observations had missing data (primarily due to suppressed county-level suicide counts in small counties) and up to 19% of UC Health observations had missing data (primarily due to suppressed self-harm counts among people aged 60+). All statistical analyses were done using R version 4.1.2.^40^

## Results

**Figure 1:**
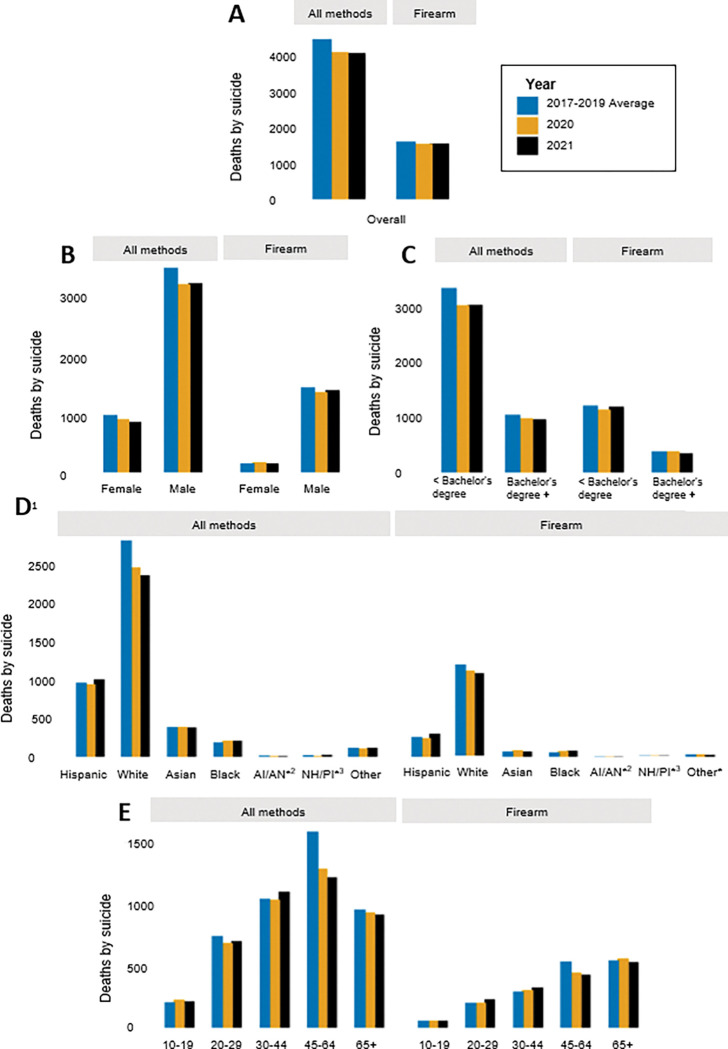
Counts of death by suicide in California from 2017–2021, by method of harm (**A**) in total population, and stratified by (**B**) sex (**C**) highest level of education (**D**) race/ethnicity, and (**E**) age group. ^1^All race/ethnicity categories besides Hispanic and Other are Non-Hispanic. ^2^AI/AN=American Indian (Native American)/Alaskan Native. ^3^NH/PI=Native Hawaiian/ Pacific Islander. *Counts of 30 or less. **Note: Y-axis scales differ across panels.

**Figure 2:**
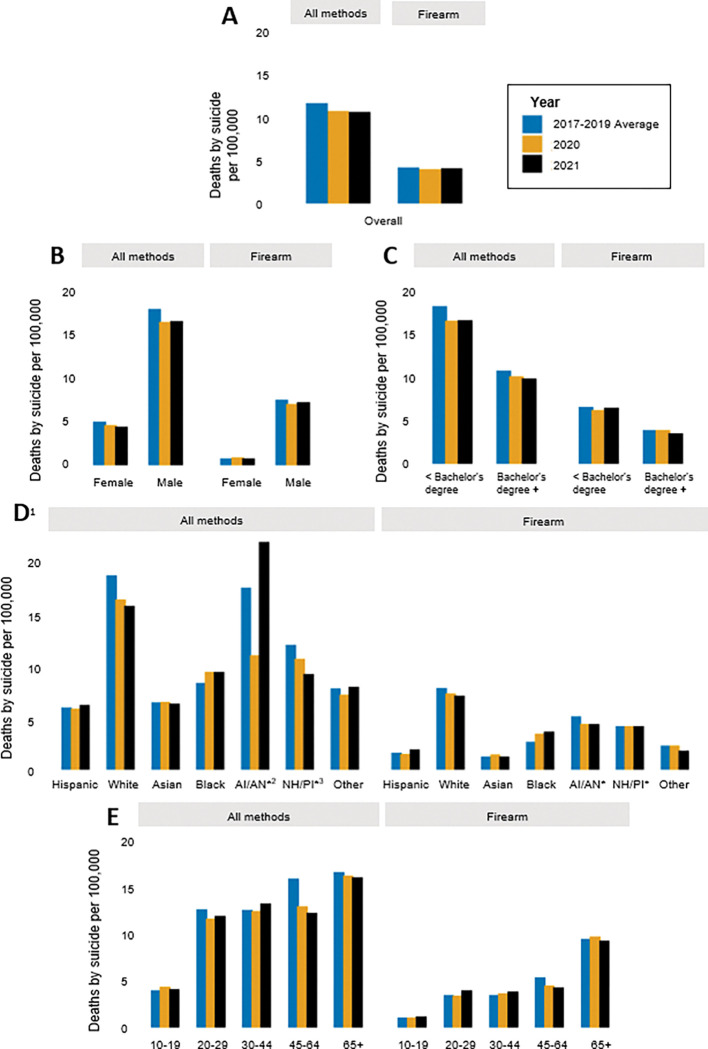
Rates of death by suicide in California from 2017–2021, by method of harm (**A**) in total population, and stratified by (**B**) sex (**C**) highest level of education (**D**) race/ethnicity, and (**E**) age group. ^1^All race/ethnicity categories besides Hispanic and Other are Non-Hispanic. ^2^AI/AN=American Indian (Native American)/Alaskan Native. ^3^NH/PI=Native Hawaiian/ Pacific Islander. *Rate is based on counts of 30 or less.

There was a total of 8,227 suicides in California in the two years following the onset of the COVID-19 pandemic: 4,123 (10.5 per 100,000) in 2020 and 4,104 (10.4 per 100,000) in 2021 (Fig. 1A & 2A; Supplemental Tables 1 & 2). Pre-pandemic, the state-wide suicide burden was higher, with an average of 4,484 deaths per year (11.4 per 100,000) in 2017–2019. Following the onset of the pandemic there was also a slight decline in firearm suicides, with 1,618 deaths (4.1 per 100,000) pre-pandemic, 1,550 deaths (3.9 per 100,000) in 2020, and 1,564 deaths (4.0 per 100,000) in 2021. Because the decline in overall suicide was greater than the decline in firearm suicides, the proportion of suicides involving a firearm (36.1% pre-pandemic) increased slightly by 1.5% and 2.0%, in 2020 and 2021, respectively (Supplemental Table 3).

Across the study period, suicide and firearm suicide counts and rates were higher among males compared to females (Fig. 1B & 2B; Supplemental Tables 1 & 2). Males consistently represented about 78% of all suicides and about 90% of firearm suicides in California. In the total population, counts and rates of suicide decreased by approximately 8% in 2020 compared to pre-pandemic; however, in 2021, females experienced a greater decline (about 12% reduction) compared to pre-pandemic than did males (about 8% reduction). The burden of firearm suicide increased slightly among females in 2020 compared to pre-pandemic, with 14 more deaths from firearm suicide among females (an increase from 0.8 to 0.9 per 100,000), while the burden decreased among males that year, with 82 fewer firearm suicides (a reduction from 7.5 to 7.0 per 100,000). Consistently across the study period, firearm use in suicide was more common among males, with firearm suicides accounting for about 42–44% of all suicides among males and 16–19% of all suicides among females (Supplemental Table 3).

The burden of and trends in suicide also differed across racial and ethnic groups (Fig. 1D & 2D; Supplemental Tables 1 & 2). Non-Hispanic white Californians, who comprise 37% of the total population, had the highest number of suicides, accounting for over half of all suicides and firearm suicides in the state in all years of the study period. Hispanic Californians consistently accounted for the next largest number of suicides and firearm suicides, followed by Asian, Black, Other, AI/AN, and NH/PI Californians. While rates of suicide and firearm suicide were generally highest among white Californians, AI/AN, NH/PI, and Black Californians experienced the next highest rates. Hispanic and Asian Californians had the lowest rates of suicide and firearm suicide across the study period.

The state-wide decrease in suicide following the onset of the pandemic was not felt or distributed evenly across racial and ethnic groups. For instance, compared to pre-pandemic, non-Hispanic white Californians experienced 348 fewer suicide deaths (a reduction from 19.4 to 17.0 per 100,000) in 2020 and 445 fewer (a reduction from 19.4 to 16.4 per 100,000) in 2021. In contrast, Black Californians experienced 23 more deaths (an increase from 8.7 to 9.8 per 100,000) in 2020 and 2021, compared to years prior. Though modest, Black Californians also had the highest and most stable increase in firearm suicides following the onset of the pandemic, with 17 more deaths (an increase from 2.8 to 3.6 per 100,000) in 2020 and 21 more deaths (an increase from 2.8 to 3.8 per 100,000) in 2021, compared to other racial/ethnic groups, all of whom experienced a decrease or no change in at least one of the years.

The burden of and changes in suicide and firearm suicide varied by age group (Fig. 1E & 2E; Supplemental Tables 1 & 2). In all years of the study period, Californians aged 30 to 64 accounted for the most suicide deaths (56–58%), while Californians aged 45 to 65 + accounted for the most firearm suicide deaths (61–66%). Rates were higher with increasing age, with people aged 10 to 19 having the lowest rates of suicide (4.0–4.4 per 100,000) and firearm suicide (1.1–1.2 per 100,000), and people aged 65 + having the highest rates of suicide (16.1–16.7 per 100,000) and firearm suicide (9.3–9.8 per 100,000). In 2020 however, young people aged 10 to 19 experienced 21 more suicides (an increase from 4.0 to 4.4 per 100,000) compared to pre-pandemic, while all other age groups saw a decline. The largest decline was among people aged 45 to 64, who had nearly 300 fewer suicides (a reduction from 16.0 to 13.0 per 100,000) in 2020 compared to pre-pandemic. People aged 65 + who died by suicide were consistently the most likely to use a firearm (Supplemental Table 3). Compared to pre-pandemic, the largest increase in the proportion of suicides that involved a firearm was among people aged 65 + in 2020 (from 57.0–60.2%) and among people aged 20 to 29 in 2021 (from 27.4–33.1%).

**Figure 3:**
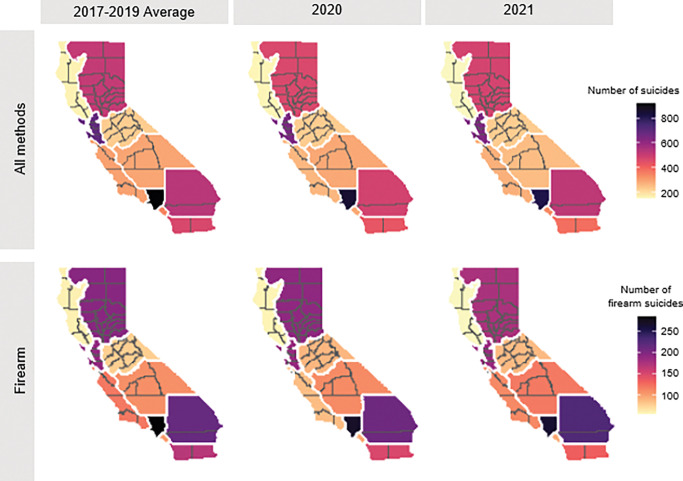
Counts of suicide, per CA census region, from 2017–2021.

**Figure 4:**
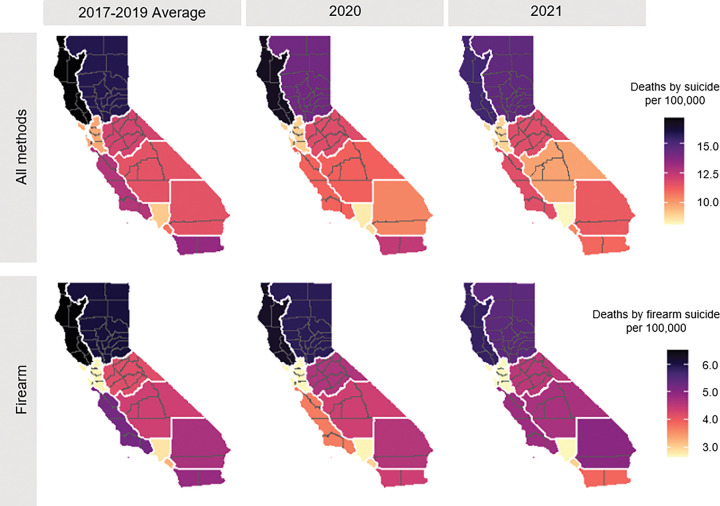
Rates of suicide, per CA census region, from 2017–2021.

Rates and counts of suicide and firearm suicide varied regionally throughout the study period (Figs. 3 & 4; Supplemental Table 4). The highest rates were consistently in the northern-most regions: Superior California and the North Coast. The lowest rates, yet the highest counts, occurred in the San Francisco Bay Area and Los Angeles County. No region saw an increase in counts or rates of suicide overall following the onset of the pandemic; however, the Northern and Southern San Joaquin Valley regions and Inland Empire region saw an increase in counts and rates of firearm suicide in 2021 compared to pre-pandemic. In addition, almost all regions had an increase in the proportion of suicides that involved firearms in 2020 and 2021 compared to years prior (Supplemental Table 3).

**Figure 5:**
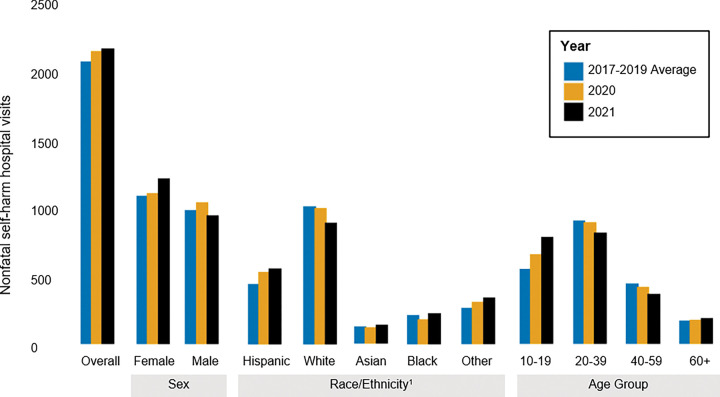
Counts of nonfatal intentional self-harm visits to UC hospital from 2017–2021, by gender, race/ethnicity, and age.^1^All race/ethnicity categories besides Hispanic and Other are Non-Hispanic.

**Figure 6:**
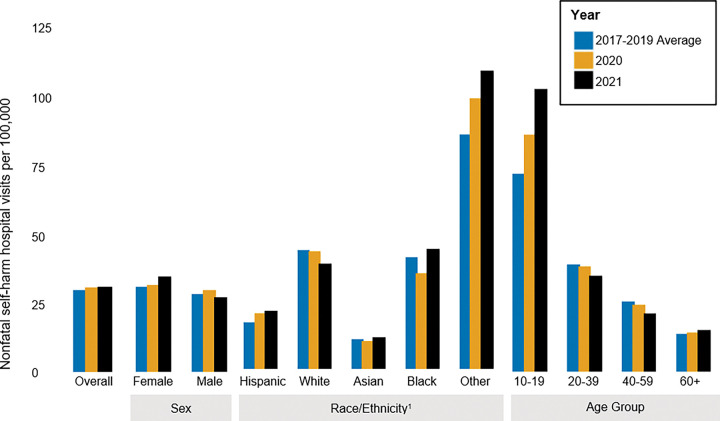
Rates of nonfatal intentional self-harm visits to UC hospital from 2017–2021, by gender, race/ethnicity, and age.^1^All race/ethnicity categories besides Hispanic and Other are Non-Hispanic.

Unlike the overall trends in suicide, the count and rate of intentional, nonfatal self-harm in UC hospitals increased following the onset of the pandemic (Figs. 5 & 6; Supplemental Table 5). The number of visits increased from an average of 2,083 per year (29.6 per 100,000) pre-pandemic, to 2,160 (30.7 per 100,000) in 2020, and 2,175 (30.9 per 100,000) in 2021. However, the burden and rate of nonfatal self-harm decreased among people aged 20–59 and white Californians in 2020 and 2021. In contrast, young people aged 10 to 19 saw the largest increase in self-harm rates, with a relative increase of 20.0% in 2020 (an increase of 553 to 663, or 70.5 per 100,000 to 84.5 per 100,000) and of 43.3% in 2021 compared to pre-pandemic (an increase of 553 to 792, or 70.5 per 100,000 to 100.9 per 100,000), with females largely driving this rise (Supplemental Figs. 2 & 3; Supplemental Table 6). Finally, the rate of nonfatal self-harm visits increased most substantially among Hispanic Californians.

## Discussion

During the first two years following the onset of the COVID-19 pandemic, the incidence of suicide declined state-wide in California, while firearm suicide rates declined much more modestly. At the same time, the incidence of nonfatal self-harm presenting in UC hospitals increased. Differential variation by sociodemographic groups and geographic areas underlay these trends, suggesting differential exposure to or impact of pandemic-era risk and protective factors, and a need for tailored allocation of state resources and prevention efforts.

The overall decline in state-wide suicide rates during and following the onset of the pandemic parallels similar findings from other states,^41–43^ and the slight decline in firearm suicide rates aligns with recent CDC data released indicating firearm suicide rates remained level between 2019 and 2020.^44^ In California, the overall decline was driven by meaningful reductions in suicide among the groups most burdened by suicide – male, middle-aged, and white Californians. In contrast, female, young, Black, and Hispanic Californians experienced increases in suicide or firearm suicide.

As in prior research, we found that males in California consistently had a higher risk of suicide and females consistently had a higher risk of nonfatal self-harm.^45^ However, females experienced a slight increase in firearm suicide in 2020 compared to pre-pandemic, while males experienced a decrease. These results may reflect the fact that the recent firearm purchasing surge led to uniquely high firearm ownership among groups historically less likely to own firearms (e.g., women) and may indicate a potential shift toward more lethal methods among this group.^24^

Young people (ages 10–19) in California experienced an increase in fatal and nonfatal self-harm overall in 2020 and 2021 compared to years prior, a trend mirroring national findings.^44^ This is especially concerning given that suicide is the second leading cause of death for young people in California and nationally.^46, 47^ The magnitude of the increase among young people was not shared by any other age groups, most of whom experienced a decrease in suicide and self-harm. Preventative efforts, including lethal means safety and mental health supports, should be prioritized for adolescents and young adults—who were uniquely impacted by recent social isolation, uncertainty, stress, and fear—given their stage of life and the importance of socialization for healthy development.^48, 49^

White Californians experienced substantial declines in suicide, firearm suicide, and nonfatal self-harm during the pandemic. Given the size of the white population and the magnitude of suicide burden among this group, this decrease drove the overall decline observed in the aggregated data. By disaggregating the data, we discovered unique trends across distinct communities. For instance, unlike all other racial/ethnic groups, Black and Hispanic Californians experienced the largest relative increase in suicide/firearm suicide and non-fatal self-harm, respectively, following the onset of the pandemic. These findings are consistent with studies in Maryland and Connecticut documenting an increase in suicide mortality among Black residents and a decrease among white residents in the months following the onset of the pandemic compared to earlier time periods,^43–42^ and with national, pre-pandemic trends showing a greater increase in suicidal behavior among Black Americans, particularly youth, compared to white Americans, from 1991 to 2019.^50^

It is likely the racial/ethnic disparities we identified are related, in part, to the pandemic-driven amplification of the structural inequities that shape population health in the U.S.^51^ and the attrition of culturally-specific factors protective of suicide. The communities most burdened by the health, economic, and social crises of 2020 and 2021 already faced disproportionate threats to their health as a result of systemic racism^8^ and other systems of marginalization that concentrate greater risk factors associated with suicide (e.g., poverty, unemployment, and mass incarceration^34, 35^) and fewer protective factors (e.g., quality education, economic development, and culturally competent mental healthcare^36–38^). Further, Black and Latino Americans, who attend church at higher rates than white Americans,^53^ may have been disproportionately impacted by the restricted ability to gather for religious worship; and religiosity has been linked to reduction in suicide risk.^32, 52^ In addition, COVID-19 increased economic and labor market disparities along racial lines^7^, which have been connected to increased risk of suicide.^54^ Finally, perceived racial discrimination, which increased during the pandemic,^52, 53^ along with disparities in death from COVID-19 and police killings,^7,55^ has also been connected to suicide risk among racially/ethnically minoritized groups.^56, 57^

Another factor potentially contributing to the increase in suicide, particularly firearm suicide, among some groups may be the firearm purchasing surge of 2020 and 2021. There is an established connection between firearm access and risk of firearm suicide,^25, 58–60^ and surges in firearm purchasing, which California experienced at the onset of the pandemic, are associated with increases in firearm violence.^61, 62^ Further, a national study found that pandemic-era firearm purchasers were more likely to experience suicidality than non-owners and pre-pandemic purchasers.^63^ While we did not observe an increase in number of firearm suicides following the onset of the pandemic, the increase in proportion of suicides that involve a firearm could indicate a trend toward an increasing use of firearms for self-harm, even amidst an overall decreasing trend in death by suicide. Alternatively, the fact that nonfatal self-harm increased while overall suicide decreased may point to greater use of less-lethal (non-firearm) means of suicide, which could explain the increase in proportion of suicides that involve a firearm, even if firearm use for self-harm did not increase. Future studies with more granular data on method of suicide should explore this question. Either way, investment in firearm violence prevention strategies – including education on safe storage practices and promotion of extreme risk protection orders^64^ – may help reduce risk for firearm suicide.

For white populations and others who experienced a decline in suicide during the pandemic, a few potentially protective factors introduced during this period may have buffered or modified the expected association between the stressors of 2020 and 2021 and increased risk for suicide. For instance, a sense of shared experience may have offset the lack of social interaction by creating a feeling of collective purpose.^65^ In addition, people who lived with others during the stay-at-home order may have been alone less often or under higher levels of supervision or scrutiny within their home, which may have reduced self-harm. Reductions in in-person healthcare appointments at the start of the pandemic led to the widespread adoption of telehealth, which may have increased some individuals’ access to mental healthcare.^66^ Finally, COVID-19 relief payments may have offset financial strain for some.^67^ Each of these potentially protective factors are likely differential based on one’s access to remote work and other economic protections; non-Hispanic white and male Californians are relatively advantaged in both regards,^68^ which could help explain the decline in suicide and self-harm those groups experienced. To our knowledge, this is the first study to assess incidence of firearm suicide and nonfatal self-harm across sociodemographic groups in California following the onset of the pandemic. There are, however, several limitations. First, due to data availability, we are only able to stratify deaths by suicide across one method (i.e., firearm) and we are unable to distinguish the methods used in nonfatal self-harm. As such, we cannot pinpoint the method of suicide or self-harm driving observed changes and are unable to compare fatal and nonfatal firearm self-harm trends (although the latter is rare given the lethality of firearm suicide attempts).^69^ Further, our inability to stratify death data across more than one domain restricts the nuance of our analyses. Future research should characterize risk across the intersection of multiple groups and compare changes in other methods of suicide. In addition, our nonfatal data may not generalize to the whole of California because only UC hospitals were captured in the sample, and all were located in urban areas. Rates for nonfatal data may over- or under-estimate the true burden across groups because of the inherent uncertainty in defining hospital catchment areas; however, assuming that there were no major changes in population size over our study period, relative rates across years should remain accurate. Finally, we are unable to identify the intent of nonfatal self-harm incidents and thus cannot distinguish between suicidal self-harm and non-suicidal self-injury, a prevalent condition with distinct etiology.^70^

## Conclusions

In California, the groups most burdened by suicide – males, middle-aged, and white Californians – experienced meaningful decreases in suicide following the onset of the pandemic, driving decreases in overall population rates, while females, young, Black, and Hispanic Californians experienced increases or small decreases, worsening existing health inequities. Identifying factors underlying these trends may inform our understanding of the epidemiology of suicide in various communities. Our findings highlight the need for targeted interventions addressing structural inequities, such as implementation of more social supports and provision of basic needs; suicide prevention interventions, such as improved access to quality mental healthcare, expansion of bereavement counseling, and better suicide risk assessments; and firearm safety efforts, such as trainings on safe storage practices and education about extreme risk protection orders, all of which could reduce suicide and self-harm and promote equitable opportunity for health and wellness across the state.

## Data Availability

The dataset of deaths in California generated and analyzed during the current study are publicly-available in the California Department of Public Health – Vital Records Data (Cal-ViDa) query tool repository, https://cal-vida.cdph.ca.gov/. ^28^ The data on nonfatal self-harm cases at UC hospitals are not publicly available. However, data may be available from the authors upon reasonable request and with permission of UC Health.

## Abbreviations

AI/AN: Native American or Alaskan Native
Cal-ViDA: California Department of Public Health – Vital Records Data
ICD-10: International Classification of Disease, 10th Revision
NH: non-Hispanic
NH/PI: Native Hawaiian or Pacific Islander
PTSD: Posttraumatic stress disorder
SARS: Severe acute respiratory syndrome
UC: University of California
U.S.: United States of America
